# Machine Learning Model to Predict Pseudoprogression Versus Progression in Glioblastoma Using MRI: A Multi-Institutional Study (KROG 18-07)

**DOI:** 10.3390/cancers12092706

**Published:** 2020-09-21

**Authors:** Bum-Sup Jang, Andrew J. Park, Seung Hyuck Jeon, Il Han Kim, Do Hoon Lim, Shin-Hyung Park, Ju Hye Lee, Ji Hyun Chang, Kwan Ho Cho, Jin Hee Kim, Leonard Sunwoo, Seung Hong Choi, In Ah Kim

**Affiliations:** 1 Department of Radiation Oncology, Seoul National University Bundang Hospital, Seongnam 13620, Korea; bigwiz83@gmail.com; 2 Artificial Intelligence Research and Development Laboratory, SELVAS AI Incorporation, Seoul 08594, Korea; parkukkyu@daum.net; 3 Graduate School of Medical Science and Engineering, Korea Advanced Institute of Science and Technology, Daejeon 34141, Korea; hyck9004@naver.com; 4 Department of Radiation Oncology, Seoul National University Hospital, Seoul 03080, Korea; ihkim@snu.ac.kr (I.H.K.); jh.chang@snu.ac.kr (J.H.C.); 5 Department of Radiation Oncology, Samsung Medical Center, Sungkyunkwan University School of Medicine, Seoul 06351, Korea; dh8lim@skku.edu; 6 Department of Radiation Oncology, School of Medicine, Kyungpook National University, Daegu 41944, Korea; shinhyungpark@knu.ac.kr; 7 Department of Radiation Oncology, Pusan National University Hospital, Busan 49241, Korea; drleejuhye@gmail.com; 8 The Proton Therapy Center, Research Institute and Hospital, National Cancer Center, Goyang 10408, Korea; kwancho@ncc.re.kr; 9 Department of Radiation Oncology, Dongsan Medical Center, Keimyung University School of Medicine, Daegu 42601, Korea; jhkim@dsmc.or.kr; 10 Department of Radiology, Seoul National University Bundang Hospital, Seongnam 13620, Korea; leonard.sunwoo@gmail.com; 11 Department of Radiology, Seoul National University Hospital, Seoul 03080, Korea; verocay1@snu.ac.kr; 12 Department of Radiation Oncology, Cancer Research Institute and BK21 Four—Smart Healthcare, College of Medicine, Seoul National University, Seoul 03080, Korea

**Keywords:** machine learning, glioblastoma, radiotherapy, pseudoprogression

## Abstract

**Simple Summary:**

Even after the introduction of a standard regimen consisting of concurrent chemoradiotherapy and adjuvant temozolomide, patients with glioblastoma multiforme mostly experience disease progression. Clinicians often encounter a situation where they need to distinguish progressive disease from pseudoprogression after treatment. We tried to investigate the feasibility of machine learning algorithm to distinguish pseudoprogression from progressive disease. In multi-institutional dataset, the developed machine learning model showed an acceptable performance. This algorithm involving MRI data and clinical features could help making decision during patients’ disease course. For the practical use, we calibrated the machine learning model to offer the probability of pseudoprogression to clinicians, then we constructed the web-based user interface to access the model.

**Abstract:**

Some patients with glioblastoma show a worsening presentation in imaging after concurrent chemoradiation, even when they receive gross total resection. Previously, we showed the feasibility of a machine learning model to predict pseudoprogression (PsPD) versus progressive disease (PD) in glioblastoma patients. The previous model was based on the dataset from two institutions (termed as the Seoul National University Hospital (SNUH) dataset, *N* = 78). To test this model in a larger dataset, we collected cases from multiple institutions that raised the problem of PsPD vs. PD diagnosis in clinics (Korean Radiation Oncology Group (KROG) dataset, *N* = 104). The dataset was composed of brain MR images and clinical information. We tested the previous model in the KROG dataset; however, that model showed limited performance. After hyperparameter optimization, we developed a deep learning model based on the whole dataset (*N* = 182). The 10-fold cross validation revealed that the micro-average area under the precision-recall curve (AUPRC) was 0.86. The calibration model was constructed to estimate the interpretable probability directly from the model output. After calibration, the final model offers clinical probability in a web-user interface.

## 1. Introduction

Pseudoprogression (PsPD) is a brain lesion shown in brain magnetic resonance (MR) images, mimicking progressive disease (PD) after concurrent chemoradiation (CCRT) in patients with glioblastoma [1]. The incidence of PsPD was estimated to be 36% in a meta-analysis [2]. The classification of PsPD and PD is important to avoid the inappropriate discontinuation of the standard treatment. However, the final diagnosis is based on the following changes in a series of MR imaging. Additionally, PsPD and PD have a similar contrast-enhanced presentation in T1-weighted MR images [3,4,5]. Thus, clinicians make decisions depending on the patient symptom [6], molecular profile [7], dynamic imaging [8,9,10], or functional imaging [11]. However, special imaging such as 18F-fluoro-ethyl-tyrosine (^18^F-FET)-positron emission tomography (PET) facilities or amino acid PET is not yet approved for glioma by the Food and Drug Administration [12].

In our previous study [13], we showed the feasibility of the deep learning algorithm that is combined with the convolutional neural network (CNN) and long short-term memory (LSTM) structures. The deep learning model was implemented to classify PsPD and PD in patients with glioblastoma who completed CCRT based on the standard regimen [14]. At the time of the appearance of the suspicious lesion in brain imaging, gadolinium-enhanced T1-weighted MR images and clinical factors were collected. Specifically, nine selected MR images, age, gender, molecular features, radiotherapy (RT) information, and the interval from the day of the completion of CCRT were selected as model inputs. The machine learning model was trained and tested in a dataset collected from two institutions, termed the Seoul National University Hospital (SNUH) dataset, *N* = 78). The model performance was acceptable, showing an area under the precision-recall curve (AUPRC) of 0.87.

However, the SNUH dataset was small, and there was a need to validate this model in a more extended dataset. This study aimed to test whether the previous model could be used in a new extended dataset collected from multiple institutions, termed the Korean Radiation Oncology Group (KROG) dataset (*N* = 104).

## 2. Methods

The study was approved by the ethics committee and institutional review board (IRB) of Seoul National University Bundang Hospital (IRB No. B-1710-426-105). The ethics committee/institutional review board that approved this study also waived the need for informed consent. All the methods were performed in accordance with the relevant guidelines and regulations.

### 2.1. Study Population and Definition of PsPD and PD

The inclusion criteria were as follows: patients with primary glioblastoma should receive a complete gross total resection of tumor and CCRT. At least 4 weeks after the completion of standard treatment, patients who demonstrated a single measurable contrast-enhancing lesion of any size on gadolinium-enhanced T1-weighted MRI within 80% isodose line based on the Response Assessment in Neuro-Oncology criteria [15] were included. The exclusion criteria were as follows: patients who had an enhancing lesion before CCRT, who had residual lesion at the immediate post-operative MRI, and who underwent incomplete CCRT.

Because most institutions did not perform operation or biopsy for the contrast-enhanced (CE) lesion, the classification of PD vs. PsPD was based on expert opinions. To minimize the variability in the definition of ground truth, we provided the guides of the PsPD vs. PD definition to multiple institutions. We defined the CE lesion as PD if ≥ 1 of the following conditions were met: it was a surgically confirmed recurring lesion, it had a significant uptake at the lesion of PET, or it had an increased size on follow-up MR imaging. Meanwhile, we considered the CE lesion as PsPD if ≥ one of the following were met: it was a pathologically confirmed PsPD, the lesion decreased on follow-up MR imaging, the lesion was stable for more than 120 days, or there was no significant uptake at the lesion of PET.

### 2.2. Model Structure and New Dataset Collection

**Figure 1:**
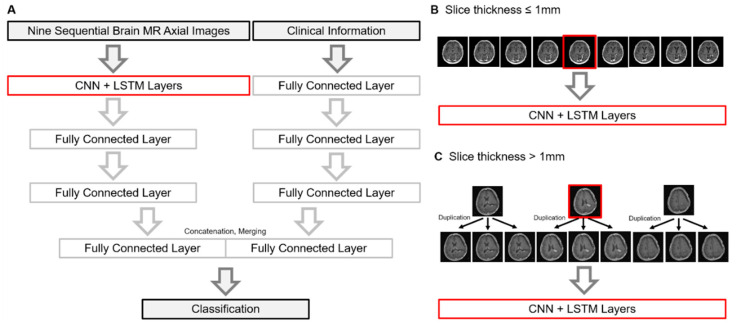
(**A**) Brief schema of the machine learning model. A graphical description how MR images were used when the slice thickness was less than 1 mm (**B**) and thicker than 1 mm (**C**), respectively. Abbreviations: PsPD, pseudoprogression; PD, progressive disease; LSTM, long short-term memory; CNN, convolutional neural network; MR, magnetic resonance.

The previous model was based on the SNUH dataset (*N* = 78). The SNUH dataset is composed of a patient’s nine axial MR images that cover the brain lesion to be classified. The slice thickness of the MR images was around 1 mm. The structure of the machine learning model is illustrated in Figure 1A. Briefly, the model is composed of two parts: CNN-LSTM for MR imaging and fully connected layers for clinical information. Firstly, the input of the CNN model is a total of nine axial gadolinium-enhanced T1-weighted brain MR images, and the output features are flattened. Flattened features are used as sequential input for LSTM layers. Secondly, clinical information is used as the input of fully connected layer, and their output was also flattened. Clinical information includes age, gender, total radiation dose, the number of fractions, interval between CCRT and the appearance of lesion, the O^6^-methylguanine-DNA methyltransferase (MGMT) methylation status, and the isocitrate dehydrogenase (IDH) mutation status. Finally, the output of the LSTM layer and the output of the fully connected layers are concatenated for the final decision. Detail activation function, kernel size, and hidden units were described in our previous study [13]. 

According to the international brain tumor imaging protocol [16], recent MR imaging for glioblastoma requires a fine slice thickness of less than 1.5 mm, and a measurable lesion should be at least 10 mm in size from axial imaging. If we selected one axial MR image containing a suspected contrast-enhanced lesion and selected three images in upward and three images in downward (total 9 images), we could capture a measurable lesion (more than 10 mm) for the model input. This measurable size was the final range for the lesions to be suspected for PD/PsPD. Furthermore, the limited numbers of images were unlikely to include irrelevant backgrounds such as the neck. We excluded other sequences of MR images, since huge resources to train/test the model were required. Further, there was a possibility for an institution not to provide the full sequence of MR imaging. Since the endpoint of this study was to develop the model from the data from multiple institutions, we minimized the requirements for the model prediction.

To test the previous model in the new extended dataset, we collected cases from multiple institutions (termed as the KROG dataset). The model network accepts MR images with a fine slice thickness of around 1 mm (Figure 1B) because nine images with a thick slice thickness (e.g., 5 mm) often cover the whole brain, neck, or other irrelevant backgrounds. However, not all the institutions have same MR protocols in terms of slice thickness. Thus, we allowed the triplicate use of three core images into nine when the image slice thickness was >1 mm (Figure 1C). This may be benefit hospitals without luxurious MR equipment to produce fine images.

### 2.3. Hyperparameter Optimization and Finalizing the Model

We tested the previous model [13] in the KROG dataset, varying the model parameters and the proportion of the dataset. We hypothesized that the both scaler range and epoch number were critical for the model, given that the SNUH dataset was outnumbered by the KROG dataset. Each parameter reflected the changes in the scaler range of clinical data and the number of epochs in terms of the previously published model. The scaler range was determined when the value of clinical information was normalized. Specifically, the scaler was ranged from −1.5 to 1.5 or from 0 to 1. Thus, the model parameters for re-training were as follows: Parameter “A” was defined as the scaler range with [0,1] and epoch number as 20, parameter “B” as [0,1] and 25, parameter “C” as [−1.5,1.5] and 20, and parameter “D” as [−1.5,1.5] and 25. Additionally, we calculated the AUPRCs as increasing the proportion of the KROG dataset from 10% to 100%. By doing this, we explored the optimal hyperparameters.

Finally, we developed the machine learning algorithm using the whole dataset (KROG dataset and SNUH dataset) with optimal hyperparameters. We performed a 10-fold cross validation and plotted the precision–recall curves. 

### 2.4. Selection of Calibration Model Selection and Implementation of User Interface

We explored the optimal calibration method by comparing three candidate calibration models: Binning strategy [17], Bayesian Binning in Quantiles (BBQ) [18], and “GUESS” model [19]. Each calibration model was trained and evaluated using 100-times repeated stratified 10-fold cross-validation. The predictions were divided into 10 bines with equal widths. Each expected calibration error (ECE) [20], root mean square error (RMSE), sensitivity, and specificity were estimated to evaluate the efficacy of the calibration models. ECE was computed as follows:

$$\mathrm{ECE}=\;\sum_{n=1}^{a}P\left(n\right)*\left|\left(r_{n}-p_{n}\right)\right|.$$

Each P(n) describes the probability of all instances that fall into bin $a_{n}$, $r_{n}$ represents the factions of true PsPD in bin $a_{n}$, and $p_{n}$ represents the mean over all predictions in bin $a_{n}$.

After calibration, the reliability diagram was depicted to establish the correlation between the model outputs and the observed probabilities.

### 2.5. Statistical Analysis

Clinicopathological characteristics were compared between patients with PsPD and PD using Student’s *t*-test and Chi-square test, or Fisher’s exact test. These statistical analyses were performed using the “Stata” version 15. The distribution of the slice thickness and validation results was represented through a violin plot, bar-plot, and heatmap using “Prism” version 8.1.2. The AUPRCs, ECE, RMSE, sensitivity, and specificity were compared among the calibration models using a repeated measure one-way ANOVA. Multiple comparison tests were also performed, and the adjusted P-value was estimated by applying the correction using Tukey hypothesis testing.

## 3. Results

### 3.1. Characteristics of KROG Dataset

**Figure 2:**
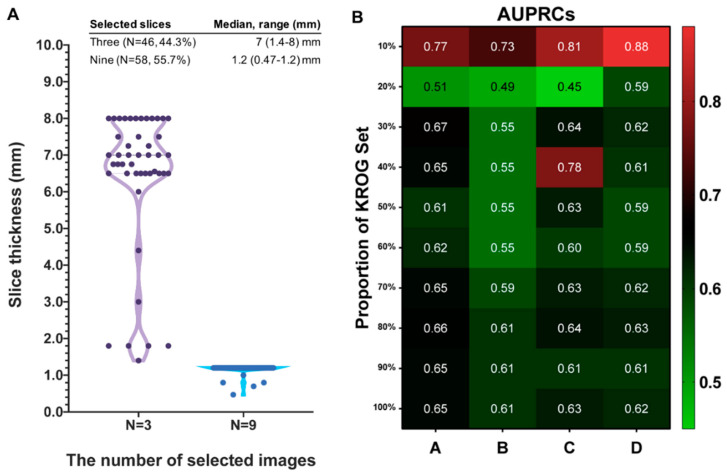
(**A**) Distribution of slice thickness between cases of the selection of 3 images for triplication and those of the selection of 9 images. (**B**) Heatmap showing the pattern of area under the precision-recall curve (AUPRC) according to the proportion of the “KROG” set with each model parameters: parameter “A” defined as the scaler range with [0,1] and epoch number as 20, parameter “B” as [0,1] and 25, parameter “C” as [−1.5,1.5] and 20, and parameter “D” as [−1.5,1.5] and 25. Abbreviations: CNN, convolutional neural network; LSTM, long-short term memory.

A total of 104 cases were collected from multiple institutions in Korea (KROG dataset). Patient characteristics are summarized in Table 1. Thirty-eight brain lesions (36.5%) turned out to be PsPD with long-term follow-up, and 66 ones (63.5%) demonstrated PD. There were no significant differences in age, gender, MGMT promoter methylation status, IDH mutational status, and the dose/fraction schedule of RT. The median interval (days) from the completion of CCRT to the appearance of brain lesion were 28 (range 19–700 days) for the PsPD group, and 95 (range 8–744 days) for the PD group (*t*-test, *p* = 0.21). For brain MR images, we allowed a policy that enables the triplicate use of three core images into nine when the slice thickness of the acquired images was > 1 mm. The median slice thickness of 46 cases (44.3%) was 7 mm (range, 1.4–8), and three MR images from each case were selected, triplicated, and used as inputs to the model. Meanwhile, 58 cases (55.7%) had a median slice thickness of 1.2 mm (0.47–1.2 mm), and were not indicated for triplicate use (Figure 2A). 

### 3.2. Testing Results of Previous Model in KROG Dataset

**Figure 3:**
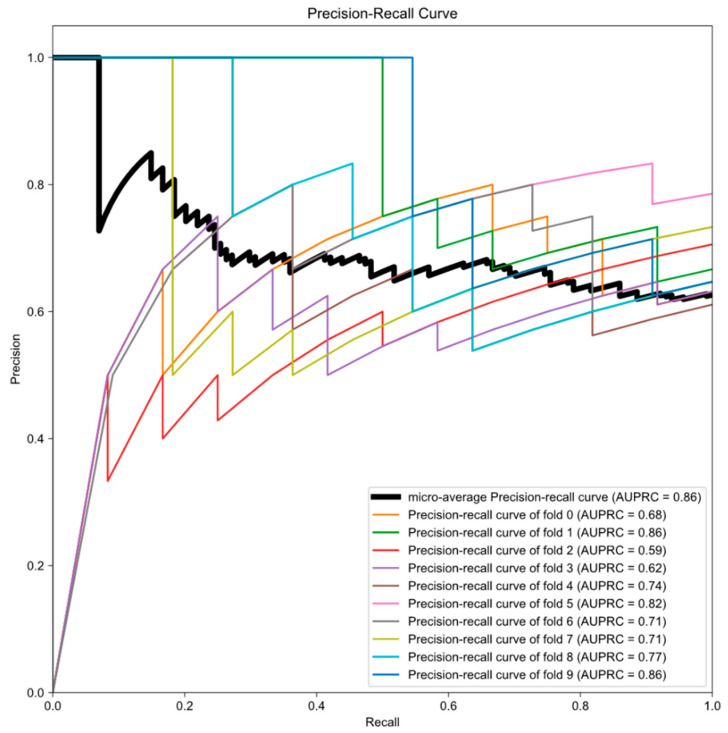
Precision-recall curves generated by 10-fold cross validation. Deep learning algorithm was the finalized optimal parameter in the whole dataset (the SNUH dataset plus the KROG dataset). Thick black line indicates the micro-average precession-recall curve, and the area under the precision-recall curve (AUPRC) is also represented in each graph.

The number of the KROG dataset is 1.3 times more than that of the SNUH dataset. Furthermore, cases with triplicated MR images were unseen data for previously published model. We hypothesized that epoch number and scaler range were critical for previously published model to predict unseen data. Thus, we tested the previous model with the KROG dataset, varying the epoch number and scaler range that are required for processing clinical data. Additionally, we tested each parameter by varying the proportion of the KROG set from 10% to 100%, and estimated the AUPRC in each dataset. As the proportion of the KROG set increases, the AUPRCs decrease regardless of the parameters. The pattern of AUPRCs according to the proportion of the KROG set is demonstrated as the heatmap in Figure 2B. By doing this, we could find the optimal hyperparameter: the parameter ‘D’. Then, we trained the model network in the whole dataset (the KROG dataset and the SNUH dataset, *N* = 182). The 10-fold cross validation was performed, and the value of the micro-average AUPRC was 0.86 (Figure 3). Thus, we finalized the model.

### 3.3. Establishment of Final Model with Calibration

**Figure 4:**
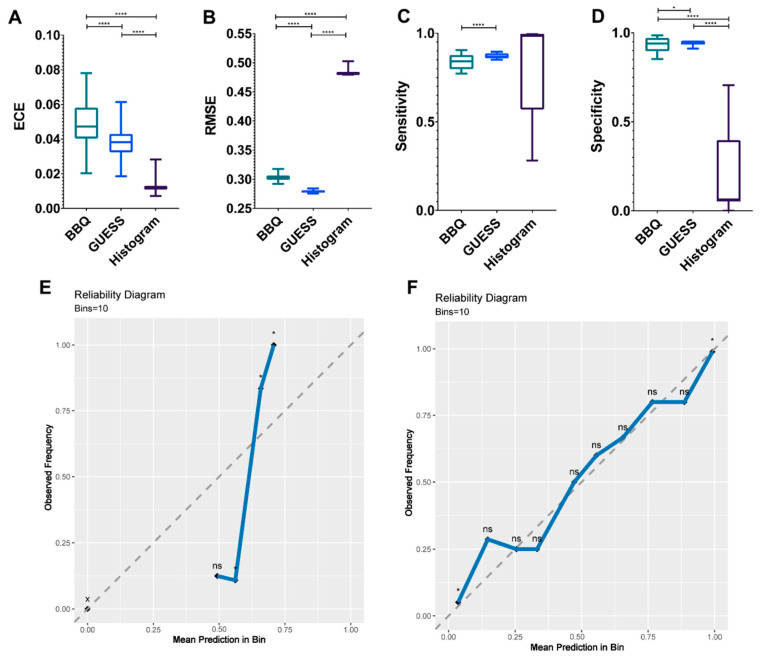
Bar plots to compare the discrimination index, including the expected calibration error (ECE) (**A**) and the root mean square error (RMSE) (**B**) according to three calibration models. Sensitivity (**C**) and specificity (**D**) are compared among the three models. Each plot represents the minimum, maximum, and mean value. Reliability diagram before calibration (**E**) and after calibration using the ‘GUESS’ model (**F**). The “x” axis represents the prediction score and the “y” axis indicates the observed frequency that is the probability of pseudoprogression (PsPD). Ten bins are used as the reliability plot. A binomial test is performed to determine the statistical significance of class distributions. **** *p* < 0.0001, * *p* < 0.05. x, empty bin; ns, non-significant. *p* values (**A**–**D**) were estimated by a repeated measure one-way analysis of variance test with multiple comparisons. Abbreviations: BBQ, Bayesian Binning in Quantiles.

Using the finalized model in this study, we sought to find the optimal calibration model by comparing the efficacy of BBQ, “GUESS”, and histogram binning. There was a significant difference in the ECEs of the calibration models (*p* < 0.0001, Figure 4A). The mean ECEs of BBQ, “GUESS”, and histogram binning were 0.049, 0.038, and 0.012, respectively. In terms of RMSE, “GUESS” showed a lower error than other models (*p* < 0.0001, Figure 4B). The mean RMSEs of BBQ, “GUESS”, and histogram binning were 0.303, 0.279, and 0.482, respectively. Sensitivity did not differ among the calibration models (*p* = 0.094); however, multiple comparisons showed that “GUESS” performed better than BBQ (0.87 vs. 0.84, adjusted *p* < 0.0001, Figure 4C). A significant difference in specificity was observed, and “GUESS” was the more superior than other models (*p* < 0.001, Figure 4D). The mean specificities of BBQ, “GUESS”, and histogram binning were 0.933, 0.945, and 0.203, respectively.

Consequently, we selected the “GUESS” as the optimal calibration model. Using the “GUESS” model, we plotted the reliability diagram before (Figure 4E) and after (Figure 4F) calibration. After calibration, we found that the observed frequency of PsPD was not significantly different from the mean prediction.

### 3.4. Examples of Correct and Incorrect Cases

**Figure 5:**
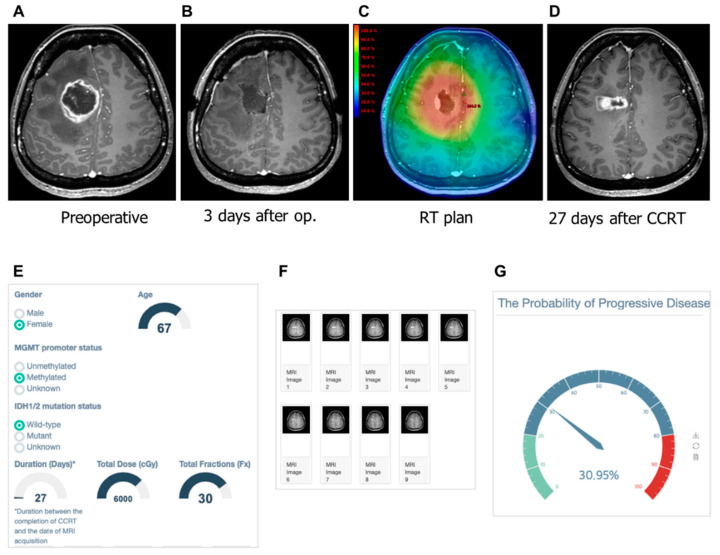
Gadolinium-enhanced T1-weighted magnetic resonance (MR) images from a 45-year-old woman with glioblastoma and the clinical application of the machine learning model. (**A**) Pre-operative MR image showing an enhanced lesion. (**B**) No residual enhancing lesion in the cavity after the gross total resection. (**C**) Radiation therapy plan image showing the isodose line. (**D**) Enhancing lesion appeared in the resection cavity within the 80% isodose line after the completion of concurrent chemoradiation. (**E**) The screenshot of clinical information is given to the web platform. (**F**) Nine MR images are selected and uploaded to the platform. (**G**) Gauge figure representing the probability of progressive disease.

**Figure 6:**
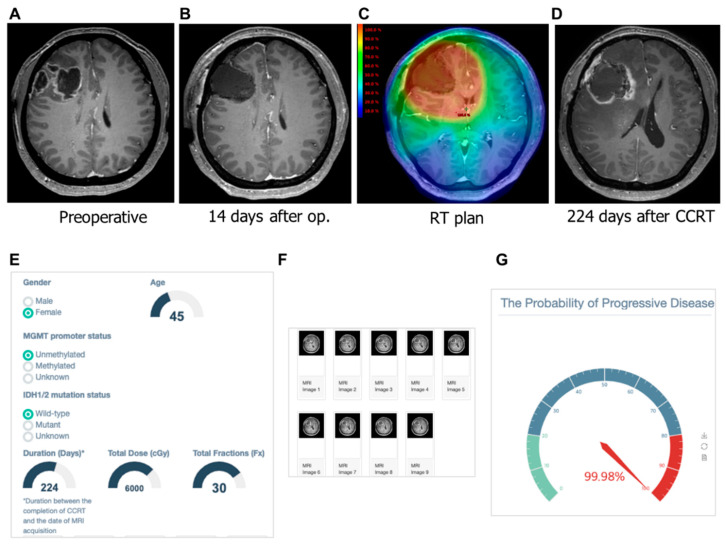
Gadolinium-enhanced T1-weighted magnetic resonance (MR) images from a 67-year-old woman with glioblastoma and the clinical application of machine learning model. (**A**) Pre-operative MR image showing an enhanced lesion. (**B**) No residual enhancing lesion in the cavity after the gross total resection. (**C**) Radiation therapy plan image showing the isodose line. (**D**) Hemorrhagic lesion as well as enhancing lesion appeared in resection cavity within the 80% isodose line after the completion of concurrent chemoradiation. (**E**) The screenshot of clinical information is given to a web platform. (**F**) Nine MR images are selected and uploaded to the platform. (**G**) Gauge figure representing the probability of progressive disease. This patient underwent a re-operation for bleeding control and resection. Pathologically, the enhancing lesion was recurrent glioblastoma.

We tested the correct and incorrect prediction by the developed model. In Figure 5, our machine learning model predicted a low probability of PD (30.95%) in patients with a contrast-enhanced lesion that appeared on the 27th day after CCRT. On the 319th day after CCRT, she had no symptoms with ongoing treatment change. As shown in Figure 6, a female 45-year old demonstrated a contrast-enhanced lesion in the right frontal lobe on the 224th day after CCRT. The machine learning model predicted this lesion as the PD with a probability of 99.98%. This patient underwent surgical resection due to bleeding in the surgical cavity. The pathologic review of the surgical specimen was recurring glioblastoma.

**Figure 7:**
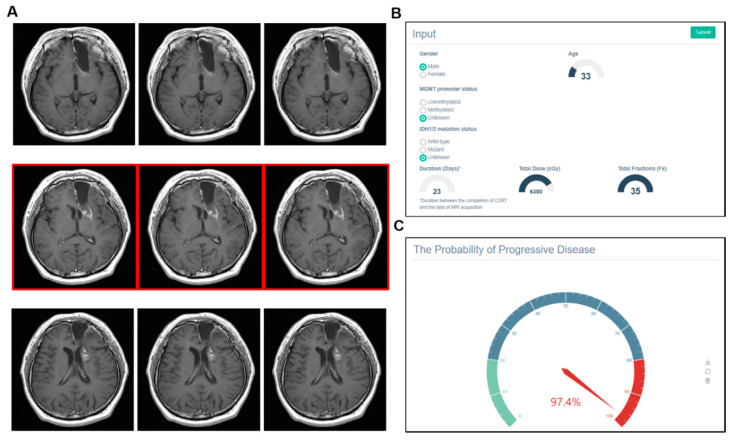
Example of incorrect prediction by the model. (**A**) Triplicate use of MR images was applied due to the thick slice thickness (>1 mm). (**B**) Statuses of the MGMT promoter and IDH were unknown. (**C**) Model predicted a contrast-enhanced lesion from the left genu of the internal capsule as progressive disease with a high probability (97.4%). However, pseudoprogression was the actual diagnosis.

In contrast, machine learning could give a wrong answer. As shown in Figure 7, a male patient who was 33 years old received CCRT with doses of 63 Gy in 35 fractions to the left frontal lobe. After 23 days, a contrast-enhanced lesion was shown from the left genu of internal capsule. Our machine learning model predicted this lesion as PD with a 97.4% probability. However, the ground truth was PsPD. Note that the MGMT and IDH statuses were unknown and the triplicate use of MR images was applied due to the thick slice thickness (>1-mm).

## 4. Discussion

In the previous study, the deep learning model showed the feasibility in two independent small datasets. We hypothesized this model could be used in the extended dataset derived from multi-institutions. In the current study, direct validation resulted in limited performance. However, the model showed acceptable performance in terms of cross-validation. The finalized model was well calibrated, then implemented in web-user interface. Thus, a clinician can access and estimate their patient’s progression probability (http://radiation-oncology-lab.ml:5000).

There were several machine learning algorithms to classify PsPD and PD. Booths et al. [21] developed a support vector machine using the features derived from T2-weighted imaging, and the model showed the accuracy of 0.86 in the prospective dataset (*N* = 7). Kebir et al. [22] analyzed the features derived from ^18^F-FET-PET images from 14 patients. This unsupervised learning revealed a specific cluster associated with PsPD, showing a 90% recall and precision. In a multiparametric brain MR study [23], a support vector machine with an eight-dimensional feature vector was developed. Although there was no testing set, the AUC was 0.94. A radiomic approach is adopted in these studies.

Meanwhile, our model was based on a deep learning approach with a single MR sequence image. Most deep learning studies in glioma focus on tumor segmentation [24,25,26], the prediction of survival [27], or the prediction of molecular profiles [28,29,30]. With respect to resolving the PsPD and PD problem, our study is the first to adopt the deep learning model in glioblastoma patients to date [31,32]. Specifically, in this study, the CNN deep learning algorithm was the key structure to capture features from gadolinium-enhanced T1-weighted MR imaging. Enhancing lesion T1-weighted MR is routinely evaluated by bidimensional measurement according to the Macdonald and RANO criteria [33]. Additionally, using single gadolinium-enhanced T1-weighted MR imaging modality will expand the practical use in clinical setting. In our model, merging clinical data with single imaging modality has an advantage. There are a few machine learning studies to incorporate clinical data in neuro-oncology [32]. Instead, researchers commonly developed a machine learning model with “wide”-dimensional input recruiting multiple huge imaging modalities. This will require substantial memory usage and cause the “curse of dimensionality” problem [34]. Several data inputs are one of the obstacles to the practical usage of the model. Thus, incorporating clinical data could reduce the abundant use of imaging modalities.

Although the network structures are the same, there are several differences in dataset between the previous [13] and the current study. First, the SNUH dataset in a previous study was small compared to the KROG dataset in this study. Secondly, the previous SNUH dataset was composed of MR images of homogeneous quality, and the clinical data were centrally reviewed by a physician. Meanwhile, the current KROG dataset originated from six institutions with various MR imaging qualities. There might also be variations in the clinical data. Input sizes are the same (Nine MR images), but in this study we allowed the use of triplicate MR images and did not include irrelevant regional information such as the whole brain or neck. These differences may contribute to the limited performance when direct validation was performed. Nevertheless, the new model in the whole dataset showed an acceptable performance in terms of the 10-cross internal validation method. Thus, this result showed us that the model network structure is still valid even in a heterogeneous dataset.

To support clinical use, the calibration model was constructed to provide PD probability. Calibration is important for clinicians to interpret the output of a machine learning model. We compared three calibration models, and found that GUESS [19], as recently published, is superior to other calibration strategies. The finalized model and optimal calibration model were integrated. By using our model, clinicians can access and estimate PD probability by applying their own clinical cases. As shown in example cases, full information and fine MR images seem to be necessary for correct prediction by model. In contrast, the incorrect prediction may be induced by missing clinical information and thick slice thickness of images. Recent response criteria [33] suggest strict imaging requirements. In 2016, the World Health Organization integrated molecular characteristics for the classification of central nervous system tumors. Therefore, we speculate that there are few cases having missing or incomplete information for the model to predict incorrectly.

Multidisciplinary team estimates PD and PsPD probability based on the time interval as well as imaging findings—e.g., 90% pseudoprogression in 1–3 months or progression in 1 year after treatment. PsPD occurs most commonly within the first 3–6 months following radiotherapy [35]. However, there is no clear cut-out time point between PsPD and PD. Clinicians tend to consider PsPD as an early event and PD as a late event after treatment, because most patients eventually experience the local disease progression. It is likely that the ratio of PsPD and PD cases is time-dependent. Following these logical processes, the “time interval” factor was introduced in the model network for the first time. On the other hand, the MGMT methylation in the model is based on the several studies addressing that it is associated with PsPD [7,36]. However, this seems to be interconnected with favorable survival from the MGMT promoter status and PsPD. In the results of the AVAglio trial (bevacizumab or placebo plus radiotherapy/temozolomide for newly diagnosed glioblastoma) data, the MGMT status was not significantly different between patients with PsPD and PD [37]. Although there are conflicting results regarding the MGMT status, we incorporated the MGMT status in the model, given the its prognostic/predictive value for treatment.

This study has several limitations. First, the KROG dataset could not be centrally reviewed due to the limited access to medical records in other institutions. In addition, variations in MR image quality could not be avoided due to the different MR imaging policies in each institution. However, this variation could be used to augment training data for the machine learning model to avoid the overfitting problem. Second, we collected as many cases as possible to finalize the model in Korea; however, they were still insufficient for deep machine learning. Additionally, the model was eventually validated with a cross-validation method with a multi-institutional dataset. Collecting datasets worldwide is required for future study to confirm the model performance in a completely held-out external dataset. Third, the ground truth—that is, the discrimination between PsPD and PD—was based on the expert opinions from the multi-disciplinary review board. Thus, the clinical decision can be affected by many confounding factors such as the second-line treatment rule, the withdrawal of the 1st line of adjuvant temozolomide, and steroid intervention in the middle of treatment. Thus, the model users should be cautioned in terms of reproducibility.

## 5. Conclusions

In conclusion, we tested the feasibility of the deep learning model in the extended dataset collected from multiple institutions. Cross validation results were acceptable, then the classifier scores were successfully transformed into interpretable probabilities by the optimal calibration model. This model could be used to support decision-making processes in a multi-disciplinary board.

## Figures and Tables

**Table 1:** Patient characteristics for the KROG dataset.

KROG Dataset	PsPD (*N* = 38)		PD (*N* = 66)			Total (*N* = 104)
	*N*	%	*N*	%	*p*	
Age (median, range)	56.5 (23–75)		55 (25–76)		0.79 *	55 (25–75)
Gender					0.02 ^†^	
Female	22	57.9	23	34.8		45
Male	16	42.1	43	65.2		59
MGMT promoter status					0.40 ^†^	
Methylated	13	34.2	18	27.3		31
Unmethylated	9	23.7	24	36.4		33
Unknown	16	42.1	24	36.3		40
IDH mutational status					0.22 ^‡^	
Mutated	2	5.2	0	0.0		2
Wild-type	15	39.5	30	45.4		45
Unknown	21	55.3	36	54.6		57
Dose schedule of RT					0.65 ^†^	
Hypofractionated	2	5.3	5	7.6		7
Conventional	36	94.7	61	92.4		97
Interval (days), median (range)	28 (19–700)		95 (8–744)		0.21 *	52 (8–744)

Abbreviations: PD, progression; PsPD, pseudoprogression; MGMT, O6-methylguanine-DNA-methyltransferase; IDH, isocitrate dehydrogenase; RT, radiation therapy. * Student’s *T*-test, ^†^ Chi-square test, ^‡^ Fisher’s exact test.
